# Association between vitamin D level and pediatric inflammatory bowel disease: A systematic review and meta-analysis

**DOI:** 10.3389/fped.2023.1155004

**Published:** 2023-04-24

**Authors:** Yan-hong Sun, Dan-dan Tian, Jian-ming Zhou, Qing Ye

**Affiliations:** Department of “A”, Children's Hospital, Zhejiang University School of Medicine, National Clinical Research Center for Child Health, Hangzhou, China

**Keywords:** 25-hydroxyvitamin D, meta-analysis, pediatric inflammatory bowel disease, vitamin D, c-Reactive protein

## Abstract

**Background:**

Previous studies have reported that the incidence of pediatric inflammatory bowel disease (IBD) is related to vitamin D, but it is still unclear. This study intends to calculate the relationship between pediatric IBD and vitamin D.

**Methods:**

A comprehensive literature search from inception to January 2023 was performed in the PubMed, EMBASE, Medline, Web of Science, and Google Scholar databases. Relevant data were extracted as required and used for subsequent calculations.

**Results:**

Sixteen papers were included, and there was no significant difference between the average vitamin D level in IBD patients and healthy controls. In addition, the overall pooled results showed that C-reactive protein (CRP) was 2.65 higher before vitamin D supplementation than after supplementation [SMD = 2.65, 95% CI = (2.26, 3.04)]. Moreover, patients with IBD in remission were 0.72 higher before vitamin D supplementation than after supplementation [OR = 0.72, 95% CI = (0.52, 1.00)].

**Conclusion:**

This study suggested that there was no obvious relationship between pediatric IBD and vitamin D, while vitamin D supplementation can improve disease activity. Therefore, follow-up still needs many prospective studies to confirm the relationship between pediatric IBD and vitamin D.

## Introduction

Inflammatory bowel disease (IBD) has two main forms, Crohn's disease (CD) and ulcerative colitis (UC). IBD can be a debilitating condition to live with, yet it is largely invisible to all but the afflicted individual (1). It is considered to be a relatively new disease because it emerged only in the past 150 years. IBD occurs in children and adolescents and can adversely affect nutrition and growth in children (2). The estimated pediatric IBD incidence in Asia and the Middle East varies from 0.5 to 11.4/100,000 person years, which has seen a sharp increase in incidence over the preceding decade (3). In addition, the epidemiological trend has been consistently observed in both adult and pediatric Asian populations (3).

Recent research has indicated that the individual's genetic susceptibility, external environment, intestinal microbiome and immunodeficiencies are all involved and functionally integrated in the pathogenesis of IBD (4–6). Overall, the pathogenesis of IBD is still controversial.

In the field of IBD research, it was found that vitamin D deficiency was associated with IBD (7). Vitamin D is a lipophilic compound synthesized in the skin under the sun, and it is hydroxylated to 25-hydroxyvitamin D [25(OH)D] in the liver and further hydroxylated in the kidneys to 1,25-dihydroxyvitamin D [1,25(OH)2D], which is the active metabolite (8). Some studies have shown that vitamin D protects the gut epithelial barrier by suppressing gut epithelial cell apoptosis (9), and other studies have shown that immunological dysregulation in IBD is characterized by epithelial damage (10). Some hormones produced by the intestines, such as melatonin, can enhance the intestinal mucosal barrier and alter the composition of intestinal bacteria to regulate the immune response (11). The complexity and multiplicity of the gut microbiota play an important role in the pathophysiology of IBD by influencing the immune system, host metabolism and gastrointestinal development (12). Additionally, vitamin D has effects on both innate and adaptive immune pathways and may even play a role in promoting immune tolerance (13). In addition, vitamin D may have an important effect on gut microbiome composition and function and consequent effects on IBD clinical (14).

Vitamin D deficiency [defined as a serum 25(OH)D ≤ 20 ng/ml or 50 nmol/L] is prevalent among IBD patients (15). Vitamin D deficiency and insufficiency are common in children worldwide (16). Recent research suggested that 40.4% of 55,844 European children are vitamin D deficient, and 13.0% are severely deficient (17). Even in the United States, 50% of children ages 1%–5% and 70% of children ages 6–11 are vitamin D deficient (18). In addition, the percentage of Canadian children with vitamin D deficiency already increased to 19.4%, and the others with severe deficiency increased to 36.8% (19). Increasing evidence suggests that low levels of vitamin D are associated with important clinical parameters and outcomes in IBD patients. The possible reasons for vitamin D deficiency in IBD patients include insufficient sunlight exposure, restricted dietary intake, intestinal inflammation leading to inadequate absorption of nutrients and bile acid malabsorption (20), or as a side effect of immunosuppressive treatment with thiopurines (21). Such researchers provide supporting evidence for the relationship between vitamin D and IBD (20). Research on the mechanisms of action has provided emerging evidence that vitamin D deficiency may be implicated in disease severity (22).

In this study, we conducted a meta-analysis and systematic review to analyze the relationships between pediatric IBD and vitamin D. The results of this study may provide new insight into the cause of pediatric IBD.

## Methods

### Sources and methods of data retrieval

We performed a comprehensive literature search that included studies from inception to January 2023; the relevant studies included PubMed, EMBASE, Medline, Web of Science, and Google Scholar databases. The following terms were used to search to cover as many articles as possible: *((Inflammatory bowel disease) OR (Crohn's disease) OR (ulcerative colitis)) AND ((Vitamin D) OR (25(OH)D) OR (Cholecalciferol) OR (25-Hydroxyvitamin D) OR (Ergocalciferol) OR (Dihydrotachysterol) OR(hydroxycholecalciferol)) AND ((children) OR(child)OR (pediatric))*. Inclusion and exclusion criteria were listed according to the requirements of this study. Articles were considered to be included if the following criteria were met: (1) published as full English research articles; (2) younger than 18 years old; (3) unified definition and diagnosis of IBD; and (4) supporting data of vitamin D. Articles that did not meet the above criteria or duplicate publications were excluded. 25(OH)D is the main form of vitamin D, and 25(OH)D is used to test and represent the vitamin D level. Moreover, a 25(OH)D unit is defined as 1 ng/ml = 2.5 nmol/L, and a 25(OH)D level lower than 50 nmol/L is defined as deficiency. (The molecular weight of vitamin D is 401, according to the formula *n* = m*M, 1 ng/ml = 1,000 ng/L = 1,000/401 nmol/ml ≈ 2.5 nmol/ml.).

In this study, articles were independently screened by two authors, and both of them subsequently screened full articles. If disagreement appeared, another author evaluated the disagreement again and formed a final result after the trade.

### Article assessment

We assessed the possibility of publication bias by constructing a funnel plot of each trial's effect size against the standard error using Egger's test to assess funnel plot asymmetry; if *p* < 0.05, there was publication bias.

Risk of bias and quality assessment were assessed through the STROBE checklist for the included studies (23). In addition, the study was conducted in accordance with the Preferred Reporting Items for Systematic Reviews and Meta-Analyses (PRISMA) statement (24).

### Data extraction

Data were extracted to numbers (Apple Distribution International, USA) for effective statistical analysis. The following data were obtained from the included studies: basic characteristics, including author, published year, country, detection method of 25(OH)D and diagnostic criteria of IBD; 25(OH)D levels [mean ± standard deviation (SD)] of IBD and controls; number of individuals with IBD and controls; IBD patients in remission and C-reactive protein (CRP) changes. The criteria for remission of IBD patients were serum levels of 25-hydroxyvitamin D increased, all relevant symptoms improved, inflammatory markers were not changed, and there were no adverse events such as overdose. Finally, all data were double-checked by two authors.

### Statistical analysis

In this study, we used the standardized mean difference (SMD) to combine the mean and SD values for 25(OH)D levels. The odds ratio [OR, 95% confidence intervals (CIs)] was used to calculate the ratio of IBD patients in remission. In addition, statistical heterogeneity was assessed by Cochran's Q test and the *I*^2^ statistic. For heterogeneous results, publication bias was estimated by funnel plot and Egger's test (*P* = 0.164). The fixed-model (Mantel and Haenszel) method (if *I*^2^ ≤ 50%, *P* > 0.1) or random-model (M-H heterology) method (if *I*^2^ > 50%, *P* ≤ 0.1) was performed to obtain pooled estimates. To enhance the credibility of the results, meta-regression was used to look for potential sources of heterogeneity (Monte Carlo permutation test). All analyses were carried out using Review Manager (Version 5.3) and Stata (version 15.1).

## Results

### Basic characteristics

**Figure 1:**
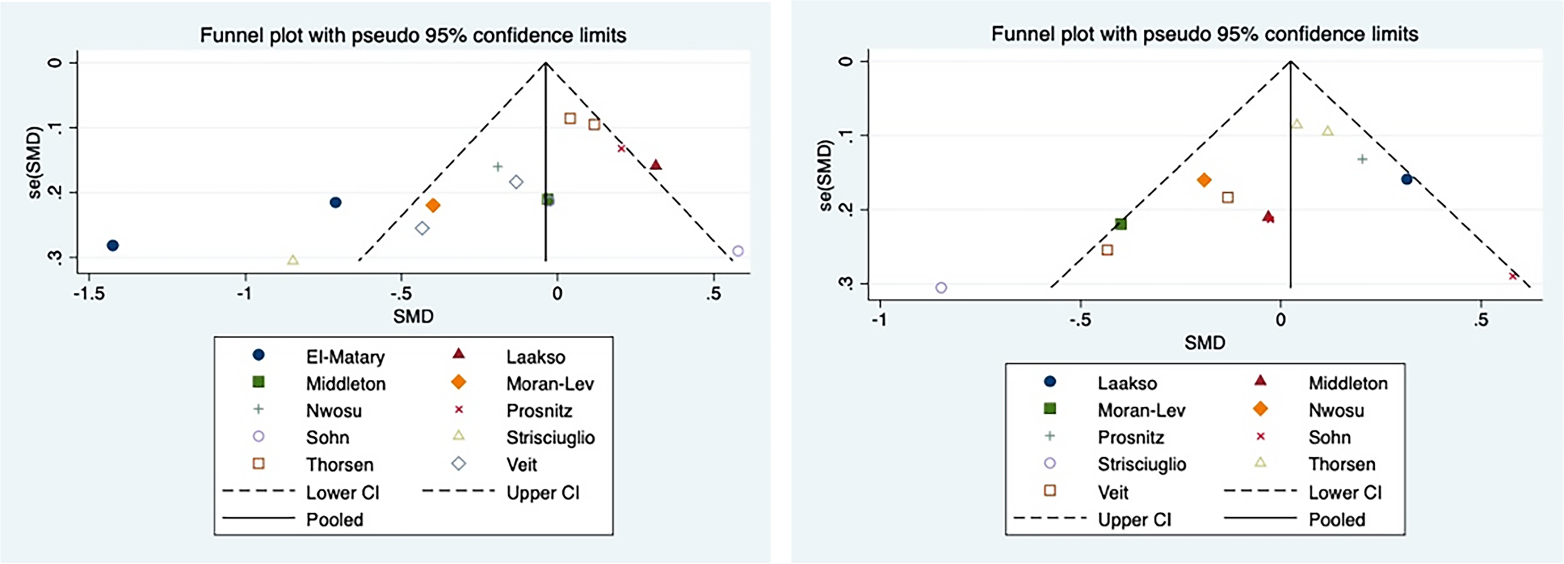
Funnel plot including (left) and excluding (right) El Matary 2011. In meta-analysis, publication bias assessment mainly depends on the Egger test. It is generally believed that there is publication bias if *p* < 0.05. In the funnel chart, it was found that one study, El Matary 2011, was far outside the funnel chart and affected the final Egger’s test results (Figure 1 left), and the Egger’s test results we used, *P* = 0.037, indicated that there was significant publication bias. After removing El Matary 2011's study, the new Egger's test was conducted on the basis of the remaining 12 studies, *P* = 0.164, indicating no significant publication bias (Figure 1 right).

**Figure 2:**
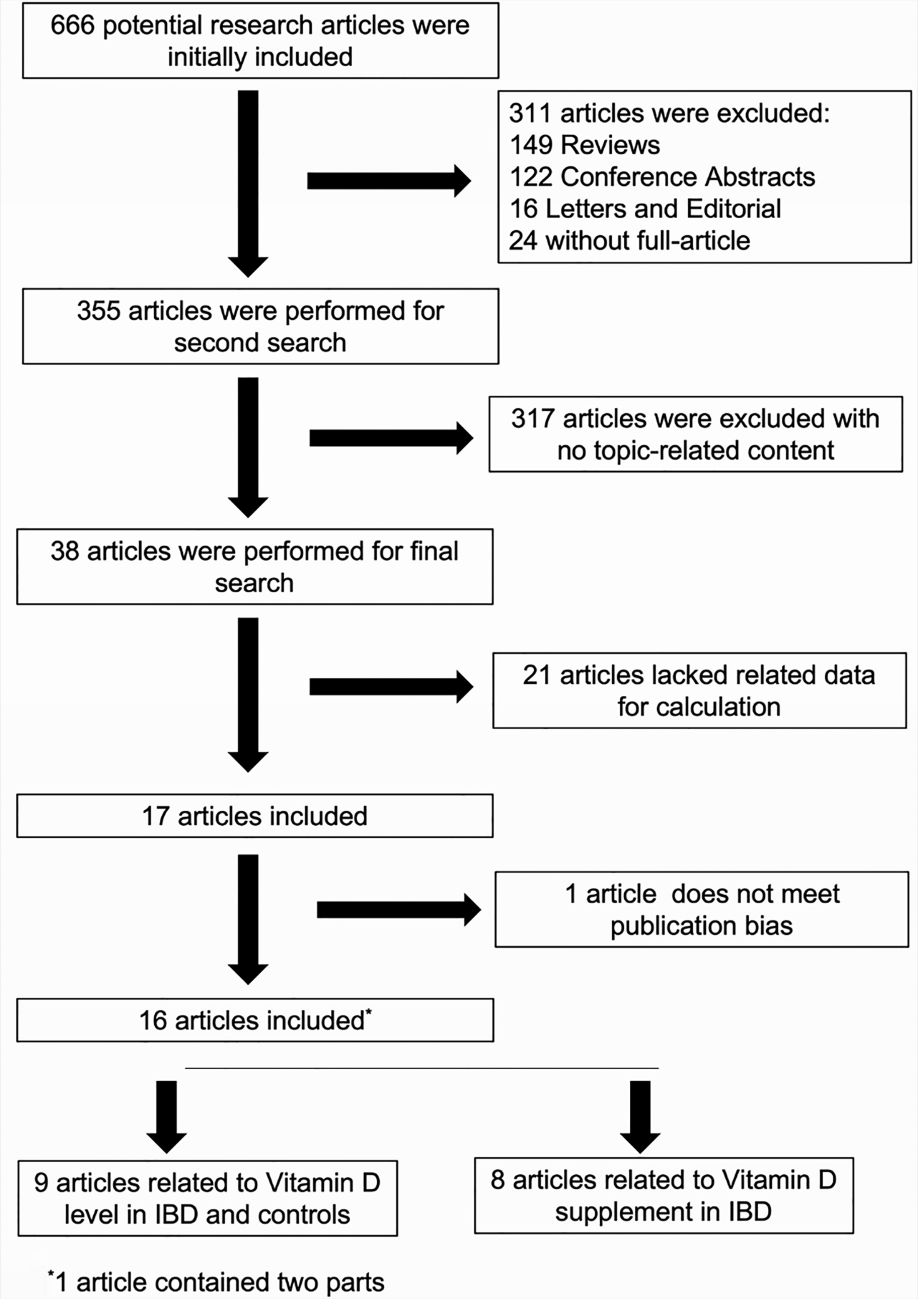
Flowchart describing the process of study selection.

In total, we searched 666 potential related articles, of which 16 papers met the inclusion criteria (19, 25–40). One of the articles from El-Matary was included in our study at the beginning but was eliminated in the later publication bias assessment (Figure 1). The flowchart that describes the process of study selection is shown in Figure 2. Nine articles reported on vitamin D levels in IBD patients and healthy controls, and eight articles reported on vitamin D supplementation in IBD patients. Of these, one article contained two parts. Detailed information on all included studies, including the type of study used, source of recruitment population, gender ratio, and age distribution, are listed in Tables 1.1, 1.2. In this study, there was no publication bias or abnormal sensitivity analysis. In addition, meta-regression showed that heterogeneity did not come from location, patient or healthy volunteers.

**Table 1.1:** Specific information on the 9 studies on vitamin D levels in IBD patients and healthy controls.

Num	Author	Year	Experimental Group			Control Group			Recruiting Population	Study Type	Type
			*N*	Gender^a^	Age(y)	*N*	Gender^a^	Age(y)			
1	Veit	2014	40	24/40	16.61 ± 2.2	116	49/116	14.56 ± 4.35	the Children's Medical Center Database of the UMass Memorial Medical Center, Worcester, Massachusetts,	case‒controlled retrospective study	CD
2	Middleton	2013	52	32/52	16.75 ± 1.75	40	15/40	10.0 ± 5.0	healthy volunteers or patients from their general gastroenterology clinic.	cohort study	CD
3	Sohn	2017	43	32/43	14.4 ± 2.8	45	26/45	13.6 ± 2.3	Seoul National University Bun dang Hospital (SNUBH)	retrospective study	CD
4	Prosnitz	2013	78	44/78	12.7 ± 2.8	221	112/221	13.5 ± 4.4	the IBD Center of the Children's Hospital of Philadelphia	cohort study	CD
5	Thorsen	2016	155	86/155	13.5 ± 1.5	384	188/384	NS	Department of Pediatrics at Hvidovre University Hospital	case- cohort study	CD
1	Veit	2014	18	15/18	16.13 ± 1.99	116	49/116	14.56 ± 4.35	the Children's Medical Center Database of the UMass Memorial Medical Center, Worcester, Massachusetts,	case‒controlled retrospective study	UC
3	Sohn	2017	17	10/17	14.5 ± 2.8	45	26/45	13.6 ± 2.3	Seoul National University Bun dang Hospital (SNUBH)	retrospective study.	UC
5	Thorsen	2016	210	96/155	14 ± 3	384	188/384	NS	Department of Pediatrics at Hvidovre University Hospital	case- cohort study	UC
6	Nwosu	2015	59	31/59	16.4 ± 2.2	116	49/116	14.6 ± 4.4	Institutional Review Boards of the University of Massachusetts and the Saint Vincent Hospital, Worcester,	cohort study	IBD
7	Moran-Lev	2019	40	20/40	13.5 ± 3.4	45	21/45	12.8 ± 3.8	Pediatric Gastroenterology Unit at Dana Dwek Children`s Hospital, Tel Aviv Sourasky Medical Center	cohort study	IBD
8	Laakso	2012	80	37/80	12.6 ± 7.5	80	NS	13.1 ± 5.7	Pediatric Gastroenterology at the Children's Hospital, Helsinki University Central Hospital	cohort study	IBD
9	Strisciuglio	2018	33	16/33	8.5 ± 6.5	18	10/18	9 ± 5	Pediatric Gastroenterology Unit, Department of Translational and Medical Science, Section of Pediatrics	cohort study	IBD

^a^ Gender: Proportion of males in the total population.

**Table 1.2:** Specific information on the 7 studies about the CRP levels in IBD patients and changes in remission IBD patients before and after VD supplementation.

Num.	Author	Year	N	Gender^a^	Age(y)	Vitamin D supplementation method	Recruiting Population	Study Type	Type
7	Moran-Lev	2019	18	NS	NS	4,000 units of vitamin D supplementation, daily, 2 weeks	Pediatric Gastroenterology Unit at Dana Dwek Children`s Hospital, Tel Aviv Sourasky Medical Center	cohort study	IBD
10	Hradsky	2017	55	30/55	13.7 ± 1.7	2,000 IU of cholecalciferol, daily,12month	Department of Pediatrics, 2nd Faculty of Medicine, Charles University in Prague and Motol University Hospital, Prague,	longitudinal prospective observational	IBD
11	Amrousy	2021	50	29/50	13.4 ± 1.2	2,000 IU of oral vitamin D3, daily, 6 months	Pediatric Department; Tropical Medicine Department; Faculty of Medicine, Tanta, University, Egypt	randomized double-blinded controlled clinical	IBD
12	Martin	2,018	23	17/23	3–16	single dose of oral cholecalciferol 100,000 to 800,000 IU	Department of Pediatrics, University of Otago Christchurch, Christchurch, New Zealand	interventional trials	IBD
13	Shepherd	2,015	76	45/76	13.1 ± 3.0	200,000 400,000 800,000 IU Cholecalciferol dose, using an age-based dosing schedule	IBD clinic at Sydney Children's Hospital, Randwick, Australia	cohort study	IBD
14	Pappa	2012	61	38/61	5–21	vitamin D_2_, 2,000 IU daily vitamin D_3_, 2,000 IU daily vitamin D_2_, 50,000 IU weekly, 6 weeks	the Clinical and Translational Study Unit of Children's Hospital Boston	randomized controlled clinical trial	IBD
15	Wingate	2014	83	45/83	14.3 ± 2.3	400 or 2,000 IU/d vitamin D3 supplement dose, 6 weeks	the McMaster Children's Hospital and the British Columbia Children's Hospital	randomized controlled clinical trial	IBD
16	Leonard	2016	138	66/138	8–21	cholecalciferol (800 IU) and calcium (1,000 mg), daily, 12 month	the Children's Hospital of Philadelphia (CHOP)	randomized placebo-controlled trial	IBD

^a^ Gender: Proportion of males in the total population.

### Vitamin D levels in IBD patients and healthy controls

**Figure 3:**
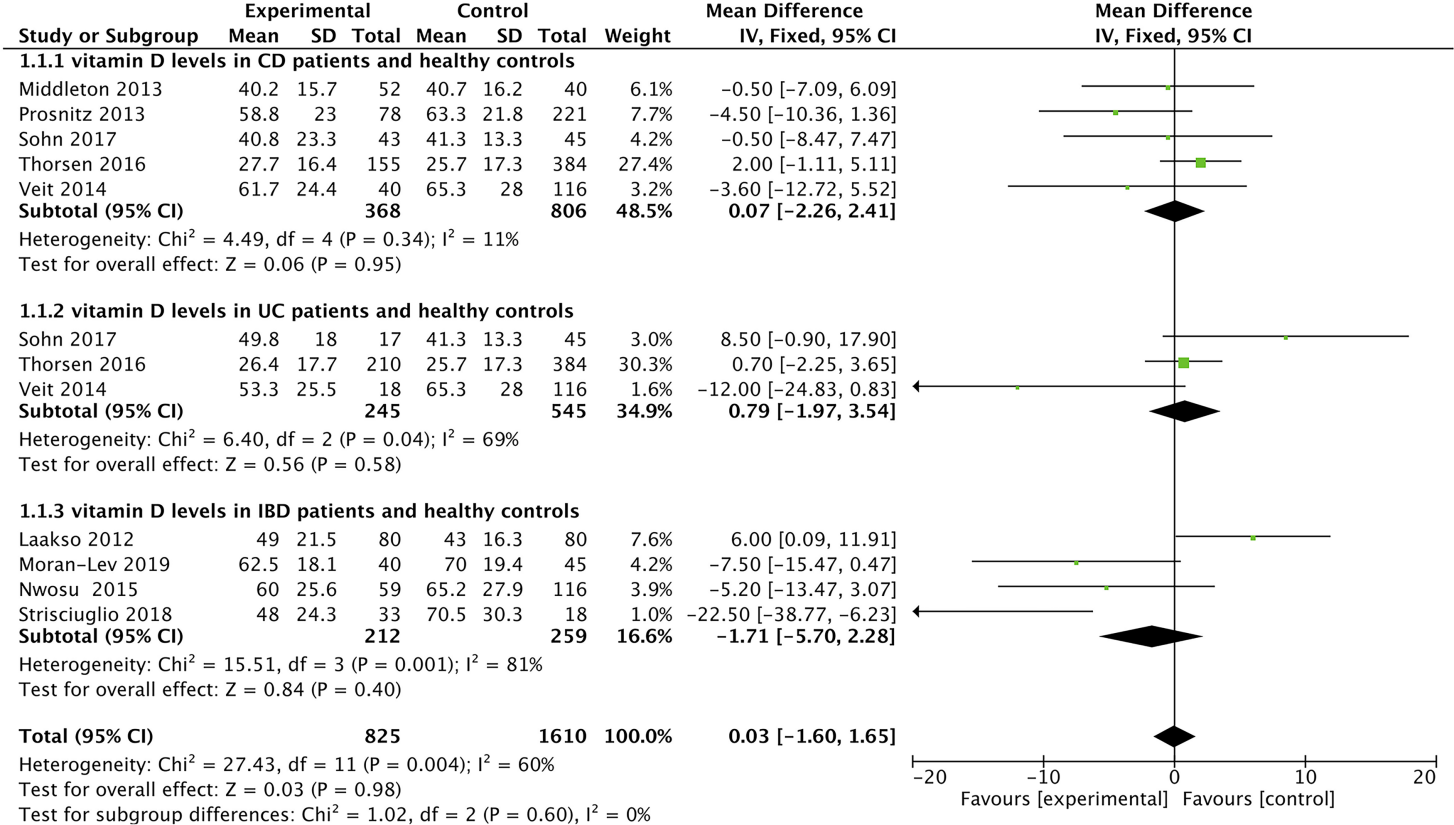
The results showed that there was no significant difference between the average 25(OH)D level in IBD patients and healthy controls [SMD = 0.03, 95% CI = (−1.60, 1.65)]. In the forest plot, there is an intersection between the rhombus and the line, indicating that the combined results are not statistically significant.

The corresponding data are listed in Table 2. Finally, 9 articles were included, containing 825 IBD patients (368 CD, 245 UC, 212 unclassified) and 1,610 healthy controls. In addition, the mean value of 25(OH)D was 48.18 nmol/L in IBD patients and 51.44 nmol/L in controls. The average 25(OH)D level was slightly higher in the control group. According to the characteristics of the enrolled patients, we conducted an overall comparison and subgroup comparison. The overall pooled results showed that there was no significant difference between the average 25(OH)D level in IBD patients and healthy controls [SMD = 0.03, 95% CI = (−1.60, 1.65)] (Figure 3). In the subgroup analysis, the results showed that the average 25(OH)D levels of CD, UC and unclassified were not significantly different compared with the control group. [SMD = 0.07, 95% CI = (−2.26, 2.41)] [SMD = 0.79, 95% CI = (−1.97, 3.54)] [SMD = −1.71, 95% CI = (−5.70, 2.28)] (Figure 3). In the forest plot, there is an intersection between the rhombus and the line, indicating that the combined results are not statistically significant.

**Table 2:** The vitamin D levels in IBD patients and healthy controls.

Author	Year	VD in pediatric IBD (nmol/L)			VD in healthy control (nmol/L)			Type
		*N*	Mean	SD	*N*	Mean	SD	
Veit	2014	40	61.7	24.4	116	65.3	28	CD
Middleton	2013	52	40.2	15.7	40	40.7	16.2	CD
Sohn	2017	43	40.8	23.3	45	41.3	13.3	CD
Prosnitz	2013	78	58.8	23	221	63.3	21.8	CD
Thorsen	2016	155	27.7	16.4	384	25.7	17.3	CD
Veit	2014	18	53.3	25.5	116	65.3	28	UC
Sohn	2017	17	49.8	18	45	41.3	13.3	UC
Thorsen	2016	210	26.4	17.7	384	25.7	17.3	UC
Nwosu	2015	59	60	25.6	116	65.2	27.9	IBD
Moran-Lev	2019	40	62.5	18.1	45	70	19.4	IBD
Laakso	2012	80	49	21.5	80	43	16.3	IBD
Strisciuglio	2018	33	48	24.3	18	70.5	30.3	IBD

VD, vitamin D; CD, Crohn's disease; UC, ulcerative colitis; IBD, inflammatory bowel disease; SD, standard deviation.

### The effect of vitamin D supplementation on IBD

**Figure 4:**
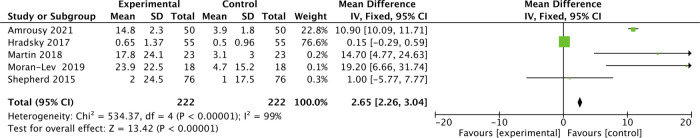
The results showed that CRP was 2.65 higher before vitamin D supplementation than after supplementation [SMD = 2.65, 95% CI = (2.26, 3.04)]. In the forest plot, there is no intersection between the rhombus and the line, indicating that the combined results are statistically significant.

A total of 5 articles described the changes in CRP after vitamin D supplementation in IBD patients, and 5 articles described changes in IBD patients who were in remission. The corresponding data are listed in Tables 3, 4. Five articles contained 222 IBD patients. The mean value of CRP was 11.83 mg/L before vitamin D supplementation and 2.64 mg/L after vitamin D supplementation. The overall pooled results showed that CRP was 2.65 higher before vitamin D supplementation than after supplementation [SMD = 2.65, 95% CI = (2.26, 3.04)] (Figure 4). In the forest plot, there is no intersection between the rhombus and the linfie, indicating that the combined results are statistically significant.

**Table 3:** The CRP levels in IBD patients before and after VD supplementation.

Author	Year	Before VD supplementation (mg/l)			After VD supplementation (mg/L)		
		*N*	Mean	SD	*N*	Mean	SD
Hradsky	2017	55	0.65	1.37	55	0.5	0.96
Moran-Lev	2019	18	23.9	22.5	18	4.7	15.2
Amrousy	2021	50	14.8	2.3	50	3.9	1.8
Martin	2018	23	17.8	24.1	23	3.1	3
Shepherd	2015	76	2	24.5	76	1	17.5

VD, vitamin D; CRP, C-reactive protein.

**Table 4:** Changes in remission IBD patients before and after VD supplementation.

Author	Year	Before VD supplementation		After VD supplementation	
		Remission	Activity	Remission	Activity
Hradsky	2017	28	27	31	24
Pappa	2012	21	3	19	4
Wingate	2014	38	50	35	46
Amrousy	2021	32	16	46	4
Leonard	2016	75	46	84	36

VD, vitamin D; IBD, inflammatory bowel disease.

**Figure 5:**
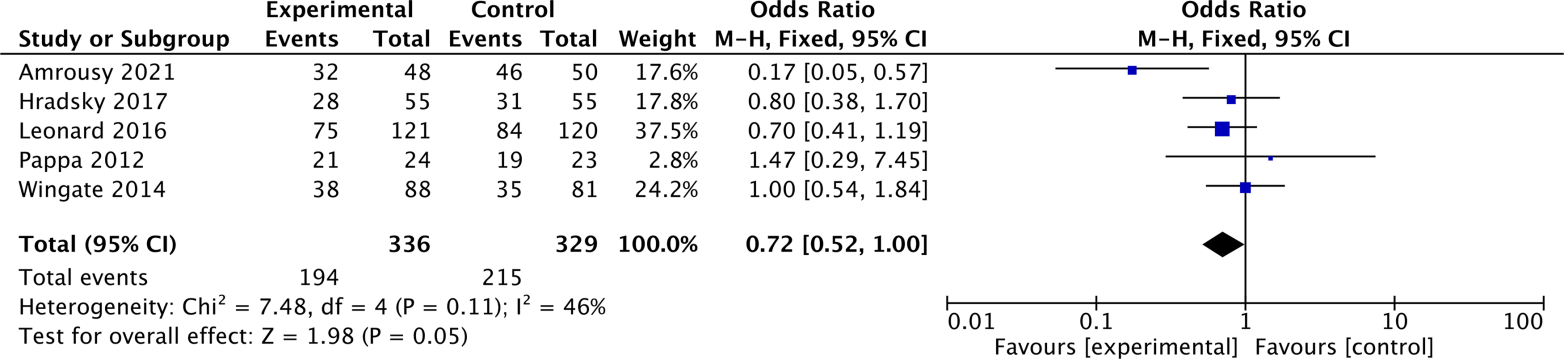
The results showed that patients with IBD in remission were 0.72 higher before vitamin D supplementation than after supplementation [OR = 0.72, 95% CI = (0.52, 1.00)]. In the forest plot, there is no intersection between the rhombus and the line, indicating that the combined results are statistically significant.

In addition, in 5 articles describing IBD in remission, the proportion of patients with IBD in remission was 57.7% before vitamin D supplementation and 65.3% after vitamin D supplementation. The overall pooled results showed that patients with IBD in remission were 0.72 higher before vitamin D supplementation than after supplementation [OR = 0.72, 95% CI = (0.52, 1.00)] (Figure 5). In the forest plot, there is no intersection between the rhombus and the line, indicating that the combined results are statistically significant.

## Discussion

In previous clinical studies, comparison results of vitamin D between pediatric IBD patients and controls were inconsistent. In this study, the results suggested that vitamin D levels were not significantly different between IBD patients and controls. However, in the other results, vitamin D supplementation decreased CRP levels and increased the proportion of patients who achieved remission. Combining these results, we hypothesized that vitamin D might be related to pediatric IBD. However, due to the existence of heterogeneity, our study did not directly reach the link between pediatric IBD and vitamin D.

Vitamin D exerts biological activity through vitamin D receptors, and VDR plays an important role in mediating the pathogenesis of pediatric IBD (42). The gene encoding VDR is recognized as a promising candidate for indicating the development of IBD (43). Robert et al. reported that vitamin D signaling contributes to innate immune responses through VDR (44). As a direct target, VDR can interfere with the inflammasome by binding to interleukin 1*β* (IL-1β) and ultimately mediate IBD (45). In addition, vitamin D induces NOD2 gene expression to regulate autotrophic function, which is also crucial for the pathogenesis of IBD (46). Therefore, there is basic research evidence for vitamin D-mediated IBD.

Vitamin D possesses strong antibacterial activity and inhibits lipopolysaccharide (LPS) to induce inflammation by downregulating inflammatory cytokines (47). In particular, vitamin D stimulates the production of pattern recognition receptors, cytokines and antimicrobial peptides, including *β*-defensins and cathepsin (48, 49). Moreover, LL-37 is the only human catalytin that has potential antimicrobial activity against both gram-positive and gram-negative bacteria, as well as some viruses (50). Indeed, cell experiments have shown that active vitamin D can inhibit the production of pro-inflammatory cytokines, including TNF-α, IL-1β, IL-6, IL-8 and IL-12, and promote the production of IL-10, an anti-inflammatory cytokine (51, 52)^.^ Low vitamin D status is often associated with systemic low-grade inflammation, as reflected by elevated C-reactive protein (CRP) levels. Linear and nonlinear Mendelian randomization (MR) analyses have been used to explore the bidirectional association between serum 25(OH)D and CRP concentrations. The observed association between 25(OH)D and CRP is likely to be caused by vitamin D deficiency, and correction of low vitamin D status may reduce chronic inflammation (53).

The microbiome and vitamin D deeply influence each other and the immune system in many different ways. The rapid corresponding immune response is activated in the innate and adaptive immune systems during hypovitaminosis D and microbiome dysbiosis (54).

This study also concluded that vitamin D supplementation was helpful in improving IBD activity, which may be closely related to the improvement of intestinal local immune function and tight junctions by vitamin D (55). However, due to the lack of sufficient prospective studies, there are also doubts about the causal relationship between the two. In the included articles, IBD treatment included vitamin D supplementation and basic treatment recommended by the guidelines. It is possible that in the course of disease treatment, as basic treatment takes effect, the damaged intestinal mucosa will be repaired, and the absorption efficiency of vitamin D will be improved, which will lead to an increase in the level of vitamin D.

This study failed to draw the results of the correlation between vitamin D and pediatric IBD. Our statistical results are the same as those of Veit but different from those of Middleton and Prosnitz. The main reasons may be related to heterogeneity and research objects, including race, age distribution, gender distribution, and so on. The main reason may be related to heterogeneity. There was obvious heterogeneity in the overall results and the subgroup analysis results. The author's analysis may have the following aspects. First, although the patients included in the study were all children, they had a large age span. Some patients are already adolescents, so the basic vitamin D level itself will be different. However, as there were not enough studies included and the included articles were not stratified for age, this study could not perform subgroup analysis based on age. Second, the regions of the patients included in the study were different. It can be seen that different dietary cultures and light intensities will also lead to different levels of basic vitamin D levels, which will also bring heterogeneity to the analysis. In addition, there were some differences in the dose and range of vitamin D supplementation, and the difference in the basic treatment plan had a greater impact on the prognosis of the disease and changes in vitamin D levels. Hormones, immunosuppressants, and biological agents have different remission rates for IBD, and the role of vitamin D supplementation in the treatment process needs to be confirmed by more prospective studies and basic studies. Finally, different vitamin D detection methods will more or less interfere with the analysis.

Although there were some shortcomings in this study, there were also some points worthy of recognition. This study systematically summarized the relationship between vitamin D and pediatric IBD for the first time, providing part of the basis for clarifying the relationship between the two.

In conclusion, this study suggested that there was no obvious relationship between pediatric IBD and vitamin D, while vitamin D supplementation can improve disease activity. We thought that the existence of heterogeneity may affect the results of this study to some extent. Therefore, follow-up still needs many prospective studies to confirm the relationship between pediatric IBD and vitamin D.
